# Detecting and identifying pathogens and antagonistic bacteria associated with *Ginkgo biloba* leaf spot disease

**DOI:** 10.3389/fmicb.2024.1346318

**Published:** 2024-02-12

**Authors:** Huoyun Shen, Xiyang Li, Zilong Li

**Affiliations:** ^1^School of Pharmacy, Guizhou University of Traditional Chinese Medicine, Guiyang, China; ^2^School of Basic Medicine, Guizhou University of Traditional Chinese Medicine, Guiyang, China

**Keywords:** Ginkgo biloba, biocontrol bacteria, Koch’s postulates, Botryosphaeria dothidea, Neofusicoccum parvum

## Abstract

**Background:**

Leaf spot disease severely impacts *Ginkgo biloba* (*G. biloba*) yield and quality. While microbial agents offer effective and non-toxic biological control for plant diseases, research on controlling leaf spot disease in *G. biloba* is notably scarce.

**Methods:**

The pathogenic fungi were isolated and purified from diseased and healthy leaves of *G. biloba*, Subsequent examinations included morphological observations and molecular identification via PCR techniques. A phylogenetic tree was constructed to facilitate the analysis of these pathogenic fungi, and Koch’s postulates were subsequently employed to reaffirm their pathogenic nature. The antagonistic experiment was employed to select biocontrol bacteria, and subsequently, the isolated biocontrol bacteria and pathogenic fungi were inoculated onto healthy leaves to assess the inhibitory effects of the biocontrol bacteria.

**Results:**

Two pathologies responsible for the leaf spot disease on *G. biloba* were identified as *Botryosphaeria dothidea* and *Neofusicoccum parvum* via the analysis of phylogenetic tree and the application of Koch’s Postulates. Additionally, we isolated two strains of biocontrol bacteria, namely *Bacillus velezensis* and *Bacillus amyloliquefaciens*. Their average inhibitory zones were measured at 4.78 cm and 3.46 cm, respectively. The inhibition zone of *B. velezensis* against *N. parvum* was 4 cm. *B. velezensis* showed a stronger inhibitory effect compared to *B. amyloliquefaciens* on the development of lesions caused by *B. dothidea* via leaf culture experiment.

**Conclusion:**

This research reports, for the first time, the presence of *B. dothidea* as a pathogenic fungus affecting *G. biloba*. Moreover, the biocontrol bacteria, *B. velezensis* and *B. amyloliquefaciens*, exhibited the capability to effectively inhibit the growth and reproduction of *B. dothidea*, indicating their promising potential as environmentally friendly biocontrol resources.

## 1 Introduction

The role of *Ginkgo biloba* (*G. biloba*) in traditional Chinese medicine is profoundly significant, with its seeds, leaves, and fruits treasured for a myriad of bioactive properties, including antibacterial, antioxidative, and cardioprotective effects (Ude et al., 2013; Zuo et al., 2017; Feng et al., 2019). Recent research has unveiled new potentials for *G. biloba* extract, such as its neuroprotective effects for retinal diseases (Martínez-Solís et al., 2019) and its application as a component in cancer treatment (Liu et al., 2022). *G. biloba* is attracting increasing attention from researchers and health enthusiasts alike (Liu et al., 2021). Its extensive medicinal potential, combined with a wide array of applications and esthetic appeal, has led to a significant increase in its cultivation and popularity across numerous countries in recent decades.

*Ginkgo biloba* has not been immune to the challenges posed by diseases, especially the debilitating impact of leaf spot disease. This issue has had a profound and detrimental effect on the widespread yield and quality of *G. biloba*, significantly affecting the production of its fruits and leaves. Furthermore, it presents a formidable barrier to the expansion of cultivation areas and the sustainability of *G. biloba* as a valuable natural resource.

Currently, *Ginkgo* diseases are controlled by pesticides. However, the conventional use of pesticides to combat these diseases has proven to be a double-edged sword, leaving behind pesticide residues that accumulate over time and foster the development of resistance in the targeted pathogens (Maji and Chakrabartty, 2014). The degradation of these residues often necessitates prolonged periods and carries the risk of considerable toxicity to humans and animals. Such resistance not only jeopardizes the sustainability of *G. biloba* cultivation but also poses a significant threat to long-term ecological balance (Guo et al., 2011).

In the pursuit of a holistic and sustainable approach to agriculture, one that harmonizes technology, ecology, and a prosperous social system, the need for biological control methods in combating plant diseases is paramount (Khan and Javaid, 2022). Biological control, being both efficient and non-toxic, offers a means to effectively preserve beneficial microorganisms in the environment, aligning with the growing demand for organic and green food products. In recent years, the agricultural sector has been increasingly embracing the use of microbial agents to combat plant diseases, making it a vital component of modern agricultural practices (Sharf et al., 2021). For example, Li et al. (2022) found *Bacillus subtilis*HAAS01, had antagonistic effect on Fusarium wilt of sweet potato, which could promote the production of endogenous hormones and resist the infection of plant diseases together with defensive enzymes and upregulation of related gene expressions. Surprisingly, despite the growing importance of *G. biloba* and the challenges posed by leaf spot disease, there is a noticeable scarcity of research specifically focusing on the biological control of the pathogenic bacteria responsible for *G. biloba* leaf spot.

This research was driven by the goal of isolating and purifying the fungus responsible for *G. biloba* leaf disease and cultivating a strain of *Bacillus* with the capacity to inhibit the growth and reproduction of this fungus. The ultimate objective is to establish a comprehensive theoretical framework for studying the causative agent behind *G. biloba* leaf spot and to explore the potential of biological control methods for its management.

## 2 Materials and methods

### 2.1 Materials and instruments

*G. Biloba* leaf samples were sourced from Guizhou University of Traditional Chinese Medicine in Guiyang, China during the months of September and October 2021. Several fungi were successfully isolated from these leaves. Antagonistic bacteria were collected from rhizosphere soil, with samples taken from a depth of 3–5 cm below the surface after removing the topsoil layer. The culture media utilized in this study included Potato Dextrose Agar (PDA) and Luria-Bertani (LB) medium. All additional biochemical reagents required for direct PCR were procured from Tsingke Biotechnology Co., Ltd.

### 2.2 Isolation and purification of fungus and antagonistic bacteria

The collected leaves underwent a series of carefully executed steps to ensure the removal of external contaminants and the isolation of endophytic bacteria. Initially, the leaves were immersed in 75% alcohol for 1 min to effectively sterilize their surfaces. Subsequently, a 2% sodium hypochlorite (NaClO) solution was applied for 3 min to further eliminate any remaining impurities. After this, the leaves were rinsed with sterile distilled water for 1 min to remove residual chemicals. The processed leaves were then dissected into 0.5 cm^2^ fragments using sterilized scissors, maintaining aseptic conditions throughout. These fragments, now devoid of external contaminants, were inoculated onto PDA and incubated at a temperature of 28 C for 2 days.

Single colony cultures that developed on the PDA medium were singled out for further investigation. Each isolated colony was assigned a unique identifier to distinguish and track individual colonies. This process was repeated through several cycles, guaranteeing the successful isolation of individual fungal colonies.

To initiate the gradient dilution, 5 *g* of soil were meticulously mixed with 45 ml of sterile water in a 250 ml conical flask. This initial mixture served as the basis for the first dilution, referred to as the 10–1 dilution. The flask underwent a rigorous shaking process for 30 min, ensuring the thorough mixing of soil and water, after which it was left undisturbed to allow for settling.

Subsequently, 1 ml supernatant was carefully transferred to a sterile glass test tube and blended with 9 ml of sterile water. This step resulted in the creation of a 10^–2^ dilution. The process of iterative dilution was repeated, each time transferring 1 ml of the previous dilution into a new test tube with 9 ml of sterile water. This series of dilutions continued until a 10^–9^ dilution was achieved.

Fifty microliters (50 μL) from each dilution were aseptically inoculated onto LB medium in triplicate. These inoculated plates were then incubated at a temperature of 37°C for a duration of 1 day. After the incubation period, individual bacterial colonies that had thrived and developed on the medium were carefully selected based on their distinctive morphological features, coloration, and other observable characteristics.

The plate confrontation method was employed to confirm and identify the purified single-colony bacteria as antagonistic microorganisms. In this procedure, the pathogenic fungus was initially inoculated onto one side of a Petri dish, with a distance of 0.5 cm from the wall, and incubated at 28°C for 24 h. Subsequently, the antagonistic bacteria were inoculated 6 cm away from the fungus on the same Petri dish and placed in a constant temperature incubator at 25°C. The diameter of the inhibition zone was measured using the criss-cross method when the pathogen colony completely covered the plate. This confirmation was essential to ensure that the isolated strains indeed exhibited antagonistic properties against the target pathogen. The experiments were meticulously conducted in triplicate to validate the reproducibility of the antagonistic effects.

Following the identification and confirmation of effective antagonistic strains, these strains were preserved by streaking them on agar slants. The slants were then stored in a refrigerator at a temperature of 4°C, ensuring the long-term viability and availability of these valuable strains for future research and applications.

### 2.3 Molecular identification of fungus and bacterial

The T5 Direct PCR Kit (Plus) and Bacterial extraction kit (Invitrogen, the USA) has been specifically tailored for the direct amplification of fungal DNA and bacterial DNA, respectively. The T5 Direct PCR Kit streamlines the process, allowing the use of these fungus samples as templates for PCR amplification. The protocol encompasses the following sequential steps:

(1) Begin by placing a 1–2 mm diameter sample into a centrifuge tube and adding 50 μL of Lysis Buffer A.
(2) Incubate the mixture at 95 C for 10 min. For more robust or challenging samples, it may be necessary to extend the lysis time.
(3) Following the incubation, vigorously shake the tube and immediately perform a centrifugation step.
(4) Collect an equivalent volume of supernatant and Dilution Buffer B, which will be crucial for subsequent steps.
(5) Utilize a volume ranging from 1 to 3 μL as the PCR amplification template, depending on the specific requirements of your analysis.

Fungal samples were subjected to PCR amplification using the common primers ITS1 and ITS4 as the upper and lower primers. Bacterial samples utilized the common primers 27F and 1492R. The PCR reaction system consisted of a 50 μL reaction volume, comprising 25 μL of 2 × T5 Direct PCR, 2 μL of the forward primer, 2 μL of the reverse primer, 2 μL of DNA template, and 19 μL of ddH_2_O.

The resulting PCR products were subsequently sent for sequencing, and the sequenced DNA sequences were compared against the NCBI (National Center for Biotechnology Information) database using Blast, an advanced bioinformatics tool, to determine the species of the isolated strains.

### 2.4 Phylogenetic tree construction

Pathogenic fungal gene sequences obtained through sequencing were subjected to thorough analysis. This analysis involved performing a BLAST comparison in the GenBank database, a renowned and comprehensive repository of genetic information. Reference strains were meticulously selected based on a stringent similarity threshold of 98% or higher. This stringent criterion ensures that the reference strains closely resemble the sequences of the pathogenic fungi under investigation.

Following the identification and selection of reference strains, a phylogenetic tree was meticulously constructed. The construction of this tree involved employing the Neighbor-Joining method, a widely recognized approach for inferring evolutionary relationships among biological entities. The process was facilitated using MEGA5.0 software.

### 2.5 The verification of Koch’s postulates for pathogenic fungus

The verification of pathogenic fungi adhered to the stringent standards of Koch’s postulates, a well-established set of criteria for establishing the causative relationship between a microorganism and a disease. In this study, Koch’s postulates were applied to healthy *G. biloba* leaves, following a protocol adapted from Pour et al. (2020).

To prepare the experimental leaves, all petioles were meticulously trimmed using sterilized scissors within a sterile environment to ensure they fit snugly into disposable plates. This process minimized the risk of introducing any contaminants or external factors that could confound the results.

Subsequently, the leaf surfaces underwent thorough cleaning with alcohol pads to achieve a high degree of surface sterilization. This step was crucial to ensure that the leaves were free from any pre-existing microorganisms that could interfere with the pathogenicity testing.

The experimental leaves were then exposed to UV radiation on their undersides for a duration of 30 min. UV radiation is a highly effective method for surface sterilization, eliminating potential contaminants that may be present on the leaf surfaces.

Autoclaved filter paper was thoughtfully placed inside the plates, and 2000 ml ddH_2_O were added to the filter paper. This step was essential to maintain suitable humidity levels within the plates, creating an environment conducive to pathogenic activity.

The UV lamp was reactivated to sterilize the upper leaf surfaces for an additional 30 min, ensuring that both sides of the leaves were effectively sterilized.

Finally, pathogenic fungi were inoculated onto the leaves using both punctured and unpunctured methods. This dual approach allowed for the assessment of different modes of infection and disease development. A control group of leaves remained untreated to serve as a baseline for comparison. Each treatment, including the control group, was meticulously replicated three times to ensure the reliability and repeatability of the results.

All disposable plates were hermetically sealed with film to prevent any external contamination during the incubation period. The plates were then placed in an incubator set at a constant temperature of 28 C, creating an environment conducive to disease development.

Leaf changes and disease symptoms were diligently monitored and recorded after a period of 14 days, allowing for the assessment of the pathogenicity of the inoculated microorganisms. Importantly, no additional ddH_2_O was introduced during this period to prevent any potential sources of external contamination, thereby upholding the integrity of the experiments.

### 2.6 The verification of Koch’s postulates for antagonistic bacteria

The leaves, having undergone the pre-treatment procedures detailed in Section 2.5., were subjected to further experimentation. To comprehensively assess the interactions between pathogenic fungi, antagonistic bacteria, and the plant, the leaves were divided into four distinct treatment groups:

The first group (1) involved leaves that were initially inoculated with pathogenic fungi. This inoculation was conducted using a puncture method, wherein the pathogenic fungi were introduced through small punctures made on the leaf surfaces. Subsequently, these leaves received an inoculation of 200 μL antagonistic bacteria.

In the second group (2), leaves were also initially inoculated with pathogenic fungi, but in this case, the inoculation was carried out without puncturing the leaves. After the pathogenic fungi had established themselves, 200 μL antagonistic bacteria were introduced.

The third group (3) comprised leaves that were solely inoculated with pathogenic bacteria, exploring the impact of pathogenic bacteria in isolation.

To serve as a baseline and control for comparison, a blank control group (CK) (4) was established. This control group received no treatment and was maintained in its original state.

Following the allocation of leaves into their respective treatment groups, all plates were carefully placed inside a constant temperature incubator set at 25 C. This controlled environment facilitated the development of interactions between the microorganisms and the plant leaves over a defined period.

The outcomes of these experiments were thoroughly documented through both photography and detailed record-keeping on the 14th day. This comprehensive approach allowed for the observation and assessment of any visible changes, disease symptoms, and the overall impact of the treatments on the plant leaves, providing valuable insights into the interactions and effects of the microorganisms under investigation.

### 2.7 Statistical analysis

Oneway analysis of variance (ANOVA) with the Least Significant Difference (LSD) test was employed for significance analysis. A significance level of *P* < 0.05 was considered. Figures were processed using Photoshop CS5.

## 3 Results

### 3.1 Isolation and purification of fungus and bacteria

**FIGURE 1:**
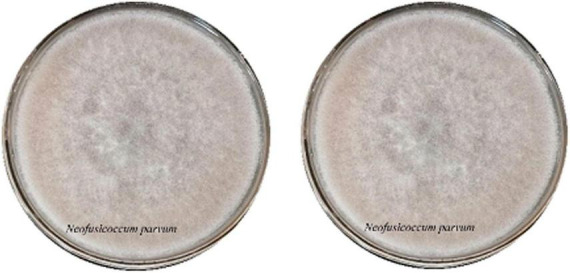
The colony of pathogen and the antimicrobial activities of antagonist against pathogen.

A total of 19 fungal strains and 5 bacterial strains were isolated from infected leaves and rhizosphere soil of *G. biloba*. The identical strain isolates were distinguished based on their morphological characteristics using both a light microscope and visual observation and identified through sequencing and genetic alignments. Among these isolated fungi, *Botryosphaeria dothodea* and *Neofusicoccum parvum* were identified as potential pathogen through checking on the reference literatures. Colonies of these two strains on PDA medium were initially white, gradually transitioned to lime green, with some eventually turning black in the later stages (Figure 1).

### 3.2 Phylogenetic tree

**FIGURE 2:**
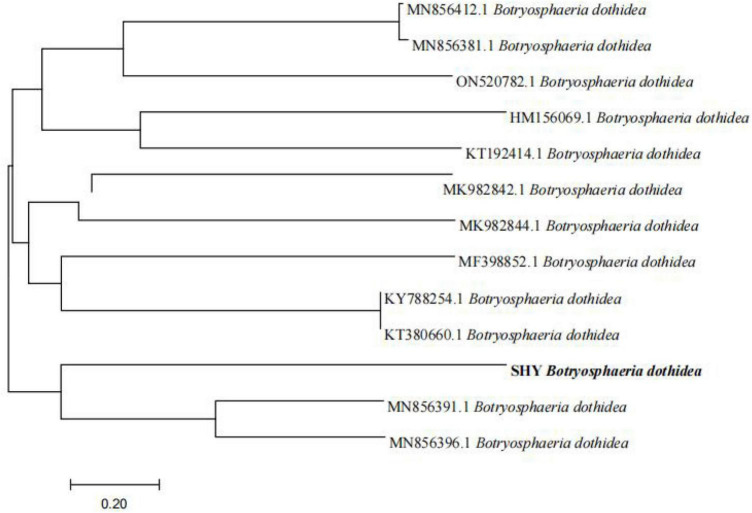
Phylogenetic tree.

A phylogenetic tree was constructed using Mega 5.0 (Figure 2) to elucidate the evolutionary relationships among isolated *B. dothidea SHY0821* and other related strains. The tree’s topology highlighted an intriguing insight into their genetic divergence. Notably, *B. dothidea* SHY0821 shares a common branch with MN856391.1 and MN856396.1, suggesting a common ancestry, however, further analysis indicated separate evolutionary trajectories. This observation strongly suggests significant genetic distinctions between SHY0821 and its counterparts within the species.

The resulting phylogenetic tree offered valuable insights into the evolutionary relatedness of the pathogenic fungi and their potential genetic relationships with the selected reference strains. This in-depth analysis is instrumental in shedding light on the genetic diversity and evolutionary context of the pathogenic fungi, aiding in the understanding of their pathogenicity and genetic characteristics.

### 3.3 Verification of Koch’s postulates for pathogenic fungus

**FIGURE 3:**
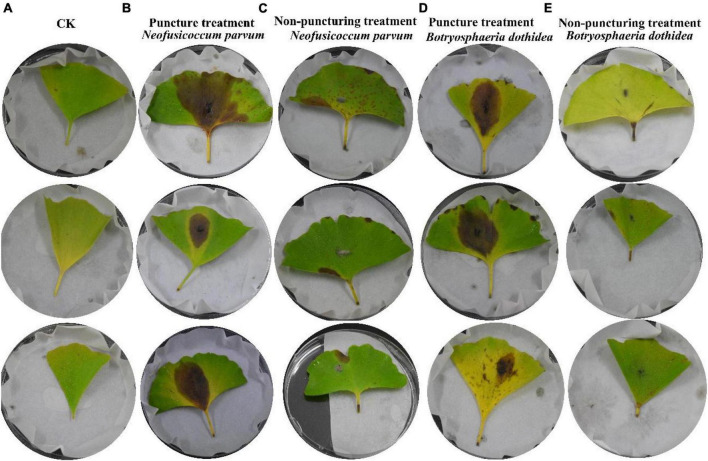
Validation of Koch’s postulates for Pathogens. Each group has 3 replicates. **(A)** The blank control (CK); **(B)** inoculation of *N. parvum* via puncture; **(C)** inoculation of *N. parvum* without puncture; **(D)** inoculation of *B. dothidea* via puncture; **(E)** inoculation of *B. dothidea* without puncture.

The experimental results detailed in Figure 3 demonstrated the successful validation of Koch’s postulates for the pathogenic fungi, *B. dothidea* and *N. parvum*, associated with *Ginkgo* leaf spot disease. The inoculation of *G. biloba* leaves with these fungi, whether through punctured or unpunctured methods, led to visible necrosis. Notably, necrosis appeared sooner in the puncture-inoculated leaves compared to the unpunctured group, suggesting a more rapid onset of symptoms due to the method of inoculation.

The disease progression was characterized by necrotic lesions, initially observed at leaf tips or margins, gradually developing into yellow to brown lesions spreading toward the mid rib. Control leaves from the same branch remained healthy, reaffirming the association of these specific fungi with the observed symptoms. Re-isolation and genetic identification of pure cultures from these lesions further confirmed the presence of *B. dothidea* and *N. parvum*, affirming their pathogenic nature in causing *Ginkgo* leaf spot disease.

Notably, *N. parvum* demonstrated a higher impact on leaf spot disease compared to *B. dothidea*. The punctured group displayed more severe symptoms, indicating a correlation between method of inoculation and disease severity (Table 1). This observation aligns with existing literature that describes *N. parvum* as a more aggressive pathogen in various plant hosts.

**TABLE 1:** Lesion diameter of fungal pathogens (cm).

CK	b	c	d	e
0 ± 0a	2.55 ± 0.78b	0.97 ± 0.26c	2.38 ± 0.59b	0.43 ± 0.24c

a,b,c: Different letters are statistically significantly (*P* < 0.05) different.

### 3.4 Isolation and purification of bacteria

**FIGURE 4:**
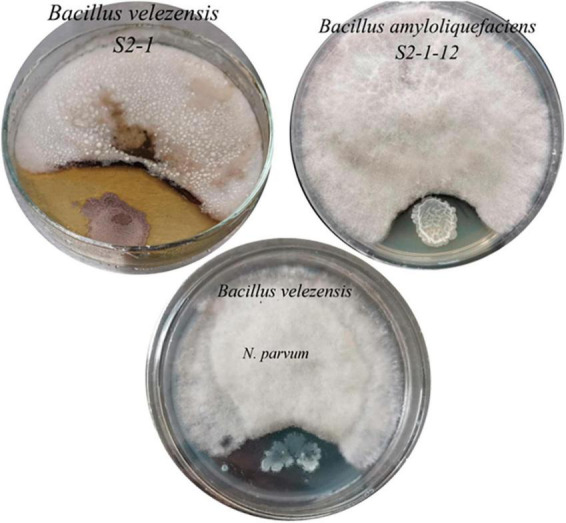
The inhibitory effect of two antagonistic bacteria on the pathogenic fungi of *B. dothidea and N. parvum*.

From the inter-rhizosphere soil samples of *G. biloba*, fourteen bacterial strains were isolated and purified. Among these strains, two displayed a significant inhibitory effect on the growth of the pathogenic fungus *B. dothidea*. Molecular analysis based on 16S rRNA identified these strains as *Bacillus velezensis* (*B. velezensis* ST2-1) and *Bacillus amyloliquefaciens* (*B. amyloliquefaciens* ST2-1-12). The average inhibitory zones measured for these strains against *B. dothidea* were 4.78 cm and 3.46 cm (Figure 4), respectively, with *B. velezensis* exhibiting a more pronounced inhibitory effect. The inhibition zone of *B. velezensis* against *N. parvum* was 4 cm (Figure 4).

### 3.5 The antagonistic bacteria’s inhibitory effect on the pathogenic fungus

**FIGURE 5:**
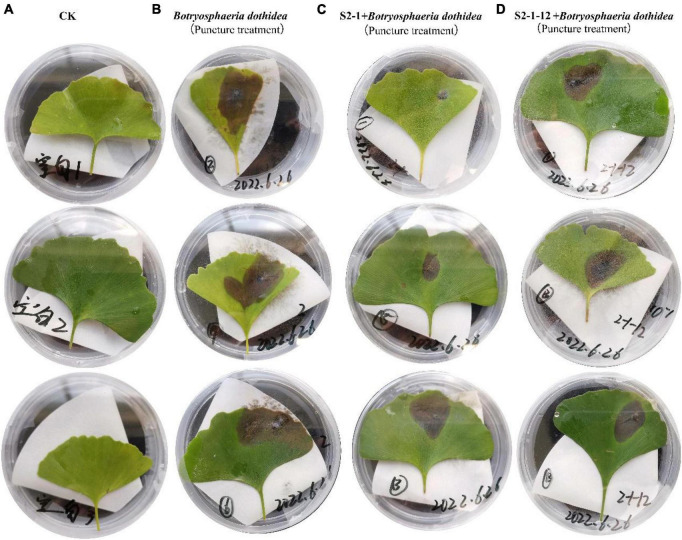
The antimicrobial effect of antagonistic bacteria on pathogens on leaf. **(A)** The blank control (CK); **(B)** inoculation of *B. dothidea* via puncture; **(C)** inoculation of S2-1+B. dothidea via puncture; **(D)** inoculation of S2-1-12+B. dothidea via puncture.

Co-inoculation of antagonist microorganisms, *B. velezensis* and *B. amyloliquefaciens*, along with the pathogenic microorganism *B. dothidea* on *Ginkgo* leaves was conducted. Initially, leaves were inoculated with *B. dothidea*, followed by the application of live bacterial suspensions of *B. velezensis* and *B. amyloliquefaciens*. After 14 days, it was observed that the leaf lesions inoculated with the antagonist microorganisms exhibited significantly smaller lesions compared to the control group solely inoculated with the pathogen (Figure 5). Additionally, the results indicated that *B. velezensis* showed a stronger inhibitory effect compared to *B. amyloliquefaciens* on the development of lesions caused by *B. dothidea* (Table 2).

**TABLE 2:** Lesion diameter of fungal pathogens (cm).

CK	b	c	d
0 ± 0a	2.88 ± 0.6b	1.33 ± 0.48c	1.95 ± 0.1c

a,b,c: Different letters are statistically significantly (*P* < 0.05) different.

*B. subtilis*, a widely distributed Gram-positive bacterium, has been previously reported to exhibit effective antibacterial properties against various pathogenic bacteria. This antibacterial effect is attributed to the secretion of various secondary metabolites, including polyketides, bacillomycin, and 3-hydroxypropionaldehyde. However, the precise mechanism by which *B. velezensis* and *B. amyloliquefaciens* inhibit *B. dothidea* requires further investigation.

## 4 Discussion

Research findings suggest that within the *Botryosphaeria* genus of the *Botryosphaeriaceae* family, both *N. parvum* and *B. dothodea* are significant fungal species. *N. parvum* has been noted for causing cankers and diebacks in various woody species globally, while *B. dothodea*, although historically perceived as a mildly pathogenic fungus, is notably prevalent and cryptic in forestry, agriculture, and natural forest ecosystems (Phillips et al., 2005; Qiu et al., 2008; Marsberg et al., 2017). *B. dothodea* has been reported in various plant diseases such as apple ring rot (Xu et al., 2014), grapevine stem wilt (Li et al., 2010), leaf spot and lesions in horticultural plants (Cunnington et al., 2007), as well as bud blight and ulcer disease in pistachios (Wunderlich et al., 2012). *N. parvum* seems to be more aggressive on important crops such as grapevine (Massonnet et al., 2017) and olive trees (Carlucci et al., 2013). The main pathogenic agent of dieback of giant sequoias, at least in Geneva, would not only be *B. dothidea*, but a close fungus of the species *N. parvum* (Haenzi et al., 2021). *B. dothidea* and *N. parvum* are, widespread, in a very large range of plant hosts, as endophytes as well as pathogens (Slippers and Wingfield, 2007; Golzar and Burgess, 2011; Park et al., 2016). This widespread distribution across diverse plant species indicates its capacity to induce leaf spots, weaken trees, reduce fruit quality, and ultimately lead to economic losses through tree death.

While it was conventionally believed that *Botryosphaeria* fungi infected plants primarily through wounds, recent studies have revealed their ability to directly invade healthy plants through natural openings such as stomata and pores (Xin et al., 2023). Koch’s postulates were employed to ascertain *N. parvum* and *B. dothidea* were responsible for *G. biloba* leaf spot disease. So far, there have been no reports regarding the pathogen responsible for leaf spot disease in *Ginkgo* trees. We isolated and identified *B. dothidea* and *N. parvum* for the first time from diseased *Ginkgo* leaves. Our results also confirmed that these two strains have the ability to penetrate through lenticels and stomata, leading to *Ginkgo* leaf disease. The variation in disease progression observed based on punctured and unpunctured inoculation implies a significant influence of the entry method on symptom onset. The ability of both fungi to penetrate *G. biloba* leaves through natural openings like lenticels and stomata supports their pathogenic nature and capability to infect the host plant.

The observed distinctions between *B. dothidea* SHY0821 and related strains could have significant implications, potentially influencing biological characteristics, ecological adaptations, and interactions with other organisms. Understanding these differences is crucial for a deeper comprehension of SHY0821’s unique attributes and potential implications. Further research is highly recommended to unveil the impact of these genetic distinctions. Investigating specific biological traits such as resistance, toxicity, metabolic pathways, or other properties in *B. dothidea* SHY0821 might provide crucial insights into its interactions with other organisms, its phylogenetic history, and the driving forces behind its evolution.

The experimental findings provided crucial insights into the pathogenicity of *B. dothidea* and *N. parvum* in causing *Ginkgo* leaf spot disease. The observations align with previous literature highlighting the pathogenic potential of both fungi across various plant hosts, both as endophytes and pathogens.

The differences in disease severity between *N. parvum* and *B. dothidea* reaffirm their varying pathogenic potentials. While historically considered marginally pathogenic, *B. dothidea* displays less aggressiveness compared to *N. parvum*, observed in various plant species like grapevines and olive trees. This discrepancy in pathogenicity might be associated with differing impacts on disease severity between the two fungi.

Given their taxonomic association within the Botryosphaeriales order, it’s reasonable to assume that *B. dothidea* and *N. parvum* share certain ecological and pathogenic characteristics. The similarity in their ecological behaviors further supports the potential for shared pathogenic traits. This emphasizes the rationale for selecting *B. dothidea* for further experiments due to its pathogenicity and ecological relevance in the context of *Ginkgo* leaf spot disease.

In conclusion, the comprehensive analysis of the experimental results confirms the pathogenic nature of *B. dothidea* and *N. parvum* in inducing *Ginkgo* leaf spot disease. The differential pathogenicity and mode of infection exhibited by these fungi underscore the importance of further research to explore their ecological, pathogenic, and taxonomic associations, aiding in the development of effective disease management strategies.

Prior studies have highlighted the efficacy of *Bacillus* species, including *B. velezensis* and *B. amyloliquefaciens*, as biocontrol agents against various pathogens in diverse crop applications. For instance, Wang et al. (2014) demonstrated the effectiveness of *Bacillus subtilis* and *Bacillus amylolyticus* in controlling apple tree rot disease post-fermentation. These findings underscore the potential of *Bacillus* species as effective biocontrol agents against plant pathogens.

The observed inhibitory effects of *B. velezensis* and *B. amyloliquefaciens* against *B. dothidea* highlight their potential as biocontrol agents. The ability of these bacteria to impede the growth of a known pathogen like *B. dothidea* underscores their antagonistic capabilities. The larger inhibitory zone of *B. velezensis* compared to *B. amyloliquefaciens* suggests a potentially stronger antagonistic effect of the former against the pathogen.

The documented effectiveness of *Bacillus* species in controlling diseases in various crops, as demonstrated by previous studies, further supports the potential of *B. velezensis* and *B. amyloliquefaciens* for controlling *Ginkgo* tree diseases. The biocontrol capabilities of these bacteria have been observed in different contexts, emphasizing their versatility and potential for disease management in various plant species. The findings underscore the significance of further research to explore and harness the antagonistic capabilities of *B. velezensis* and *B. amyloliquefaciens* against *B. dothidea*. Their potential utilization as biocontrol agents in managing *Ginkgo* tree diseases holds promise. Exploring their mode of action, field efficacy, and potential formulation as biocontrol agents specific to *Ginkgo* leaf spot disease is imperative.

The leaf inoculation experiment results validated the antagonistic effects of *B. velezensis* and *B. amyloliquefaciens* against *B. dothidea*. The reduction in lesion size in the presence of antagonist microorganisms suggests their potential in suppressing the pathogenic effects of *B. dothidea*. The superior inhibitory effect of *B. velezensis* compared to *B. amyloliquefaciens* further supports the notion of their differential efficacy against the pathogen.

The known antibacterial properties of *B. subtilis*, attributed to the secretion of various secondary metabolites, provide a foundation for understanding the potential mechanisms through which *B. velezensis* and *B. amyloliquefaciens* inhibit *B. dothidea* (Javed et al., 2021). However, the specific mechanisms by which these antagonistic bacteria impede the growth or pathogenicity of *B. dothidea* remain to be elucidated. Investigating the precise pathways, potential secondary metabolites, and their impact on the pathogen will be crucial for a comprehensive understanding.

The findings offer promising implications for the potential utilization of *B. velezensis* and *B. amyloliquefaciens* as biocontrol agents against *Ginkgo* leaf spot disease. Further research is imperative to unravel the specific mechanisms behind the inhibitory effects. Understanding these mechanisms could pave the way for more targeted and effective biocontrol strategies, ensuring the efficient management of the disease.

## 5 Conclusion

In summary, the co-inoculation experiment confirmed the inhibitory effects of *B. velezensis* and *B. amyloliquefaciens* on *B. dothidea*-induced lesions. While the antibacterial properties of related strains like *B. subtilis* offer insights into potential mechanisms, in-depth research is needed to fully comprehend how these antagonistic bacteria suppress the pathogenicity of *B. dothidea*, offering avenues for innovative disease management strategies.

## Data availability statement

The raw data supporting the conclusions of this article will be made available by the authors, without undue reservation.

## Author contributions

HS: Investigation, Writing−original draft. XL: Funding acquisition, Supervision, Writing−review and editing. ZL: Data curation, Software, Writing−review and editing.
